# Standardizing Benchmark Dose Calculations to Improve Science-Based Decisions in Human Health Assessments

**DOI:** 10.1289/ehp.1307539

**Published:** 2014-02-25

**Authors:** Jessica A. Wignall, Andrew J. Shapiro, Fred A. Wright, Tracey J. Woodruff, Weihsueh A. Chiu, Kathryn Z. Guyton, Ivan Rusyn

**Affiliations:** 1 Department of Environmental Sciences and Engineering, and; 2 Department of Biostatistics, Gillings School of Global Public Health, University of North Carolina at Chapel Hill, Chapel Hill, North Carolina, USA; 3 Department of Obstetrics, Gynecology, and Reproductive Sciences, School of Medicine, University of California, Oakland, California, USA; 4 National Center for Environmental Assessment, U.S. Environmental Protection Agency, Washington, DC, USA

## Abstract

Background: Benchmark dose (BMD) modeling computes the dose associated with a prespecified response level. While offering advantages over traditional points of departure (PODs), such as no-observed-adverse-effect-levels (NOAELs), BMD methods have lacked consistency and transparency in application, interpretation, and reporting in human health assessments of chemicals.

Objectives: We aimed to apply a standardized process for conducting BMD modeling to reduce inconsistencies in model fitting and selection.

Methods: We evaluated 880 dose–response data sets for 352 environmental chemicals with existing human health assessments. We calculated benchmark doses and their lower limits [10% extra risk, or change in the mean equal to 1 SD (BMD/L_10/1SD_)] for each chemical in a standardized way with prespecified criteria for model fit acceptance. We identified study design features associated with acceptable model fits.

Results: We derived values for 255 (72%) of the chemicals. Batch-calculated BMD/L_10/1SD_ values were significantly and highly correlated (*R*^2^ of 0.95 and 0.83, respectively, *n* = 42) with PODs previously used in human health assessments, with values similar to reported NOAELs. Specifically, the median ratio of BMDs_10/1SD_:NOAELs was 1.96, and the median ratio of BMDLs_10/1SD_:NOAELs was 0.89. We also observed a significant trend of increasing model viability with increasing number of dose groups.

Conclusions: BMD/L_10/1SD_ values can be calculated in a standardized way for use in health assessments on a large number of chemicals and critical effects. This facilitates the exploration of health effects across multiple studies of a given chemical or, when chemicals need to be compared, providing greater transparency and efficiency than current approaches.

Citation: Wignall JA, Shapiro AJ, Wright FA, Woodruff TJ, Chiu WA, Guyton KZ, Rusyn I. 2014. Standardizing benchmark dose calculations to improve science-based decisions in human health assessments. Environ Health Perspect 122:499–505; http://dx.doi.org/10.1289/ehp.1307539

## Introduction

Public health agencies [e.g., the U.S. Environmental Protection Agency (EPA) and the California EPA] conduct health assessments of environmental chemicals to determine the likelihood of human health hazard and to establish levels of exposure considered as health protective. To derive quantitative toxicity values (i.e., cancer slope factors or reference doses/concentrations) for comparison to environmental exposure levels, the relationship between a dose/concentration of a chemical and a health outcome is characterized (U.S. EPA 2012b). Data from occupational cohorts or from studies in experimental animals are typically used for this purpose [National Research Council (NRC) 1983]. The first step in developing toxicity values is identifying, for each data set, a point-of-departure (POD) dose, from which extrapolations to environmentally relevant doses are made.

PODs traditionally used in noncancer health effect assessments are no-observed-adverse-effect-levels (NOAELs) or lowest-observed-adverse-effect-levels (LOAELs) (U.S. EPA 2012b). NOAELs and LOAELs are limited to the dose groups tested in a particular study and are not informed by the shape of the dose–response relationship (Barnes and Dourson 1988; Travis et al. 2005). Benchmark dose (BMD) modeling, a process of fitting a model to dose–response data, estimates a POD that is associated with a predefined level of biological response [i.e., the benchmark response (BMR)] (Crump 1984). BMD modeling addresses some limitations of NOAELs and LOAELs in that BMDs account for the shape of the dose–response curve, are more independent of study design elements such as dose choice or spacing, and can be more easily compared across multiple chemicals. In addition, estimating the BMD lower limit (BMDL) informs uncertainty in risk estimates. However, not all dose–response data sets are amenable to BMD modeling, for example, when group sizes are very small but otherwise reflect the species of choice (as is often the case with dog studies).

BMD modeling is traditionally conducted on a chemical-by-chemical basis, with variability introduced during selection of critical end points, BMR values and models used to compute BMDs, as well as in evaluating model fit (Travis et al. 2005; U.S. EPA 2012b). For example, the biological significance of a given magnitude of change can differ among end points, especially when they range in severity. Thus, even though the choice of BMR may vary from chemical to chemical and study to study, we investigated ways to standardize BMD methodology to increase consistency in POD derivation, reduce complexity, and improve efficiency.

A large database of developmental toxicity studies was used previously to derive BMD estimates (Allen et al. 1994a, 1994b) to demonstrate that a standardized approach to dose–response modeling is advantageous. Using a limited set of data and models, it was shown that BMDs based on a 5% extra risk response were within an order of magnitude of statistically derived NOAELs. In the present study, we expand upon this previous work by applying a standardized process for conducting BMD modeling to 880 dose–response data sets for 352 environmental chemicals extracted from publicly available human health assessments. Using standard approaches, as recommended by the U.S. EPA (2012a), we evaluated multiple end points and identified features of animal study methods that may influence their utility for BMD modeling.

## Methods

*Data sets*. The U.S. EPA Integrated Risk Information System (IRIS) (U.S. EPA 2013a), the U.S. EPA Office of Pesticide Programs (U.S. EPA 2013b), the U.S. EPA Superfund Regional Screening Levels (RSL) (U.S. EPA 2013d), and the California EPA (Office of Environmental Health Hazard Assessment 2009a, 2009b) were surveyed for publicly available information on chemicals with human health assessments. Superfund RSL also included toxicity values from the U.S. EPA Provisional Peer Reviewed Toxicity Values (U.S. EPA 2013c), the Centers for Disease Control and Prevention’s Agency for Toxic Substances and Disease Registry (2013), and the U.S. EPA Health Effects Assessment Summary Tables (U.S. EPA 2011a). We collected both noncancer and cancer toxicity values [reference doses (RfDs), reference concentrations (RfCs), oral slope factors, inhalation unit risks, and cancer potency values], and PODs that were used to derive the toxicity values, where applicable (NOAELs, LOAELs, and BMDLs).

For each toxicity value, we extracted the dose–response data from the critical study used in the human health assessment. For each chemical, we obtained the name and a unique chemical identifier in the form of the Chemical Abstracts Service Registry Number (CASRN). The chemicals and their associated toxicity values, PODs, dose–response data, and calculated BMD/Ls are from the Carolina Center for Computational Toxicology (http://comptox.unc.edu/bmddata.php).

*Chemical structure curation*. Chemicals lacking CASRN were removed (e.g., mixtures such as “coke emissions”). CASRN were used to retrieve chemical structure information in the form of simplified molecular-input line-entry system codes (Weininger et al. 1989), which were converted to structure-data files using KNIME: the Konstanz Information Miner (Berthold et al. 2007). A rigorous chemical structure curation protocol (Fourches et al. 2010) was applied to ensure that the chemical structures were standardized and that mixtures and chemicals for which descriptors cannot be calculated (i.e., inorganics, organometallics) were removed.

*BMD/L calculation*. BMDs and BMDLs were calculated in a consistent fashion using BMDS Wizard (beta version 1.6.1) (ICF International 2012) and BMD Software (BMDS, version 2.3.1; http://www.epa.gov/ncea/bmds/). Specifically, we applied automated rules with no manual interpretation of results with respect to the following: *a*) selection of the BMR value, *b*) choice of the models(s), *c*) model fitting criteria, *d*) computation of the BMDL, and *e*) reporting of BMD and BMDL values. All automated rules were consistent with BMD modeling guidelines (U.S. EPA 2012a). The results are hereafter referred to as “batch-calculated” BMDs and BMDLs.

**Figure 1:**
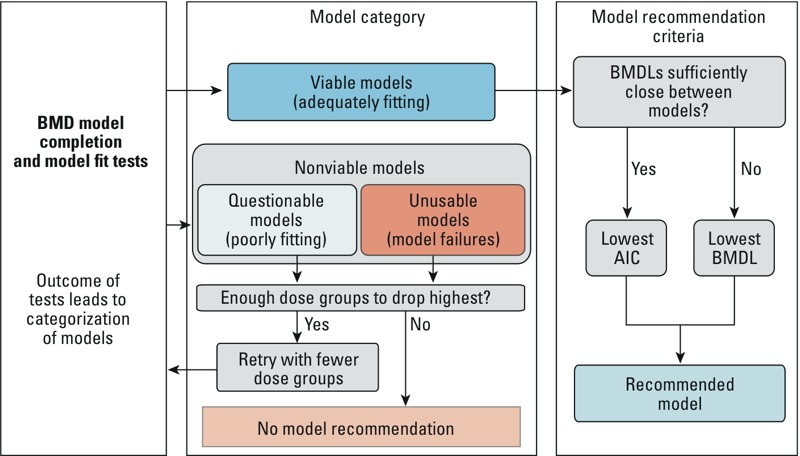
Schematic of BMDS Wizard workflow, adapted with permission from ICF International. AIC, Akaike’s information criterion.

The BMDS Wizard program (ICF International) was used to automatically run BMDS. This program also recommended BMD/Ls for the collected dose–response data, based on the best-fitting model selected according to decision logic determined prior to modeling. The model decision logic and the criteria used to determine each model’s viability, based on adequacy of the fit of the model to the data are specified in Supplemental Material, Table S1. That is, after models are fit to the dose–response data, the tests listed in Supplemental Material, Table S1 were used to assign model fits of the dose–response data to Unusable, Questionable, or Viable categories by BMDS Wizard. As described in Figure 1, only Viable model outputs are used in the remainder of this analysis. We termed such Viable models “successful.”

Data sets were grouped according to dose–response type (continuous, dichotomous, or dichotomous-cancer), which guided the choice of BMRs and the types of models used to calculate BMDs. All models specified in the BMD modeling guidelines (U.S. EPA 2012a) were run for the appropriate data type (Table 1). Several additional model types that take into account more advanced biology—such as nested dichotomous, background-dose, background-response, repeated response, concentration/time, and multi-tumor models—were not within the scope of this project.

**Table 1:** Summary of BMRs and models used in BMDS, according to dose–response type.

Dose–response type	Dichotomous	Continuous	Dichotomous-cancer
Benchmark response	10% extra risk	Change in the mean = 1 control-group SD^*a*^	10% extra risk
Models used to calculate BMDs and BMDLs^*b*^	Gamma, Dichotomous-Hill, Logistic, LogLogistic, Probit, LogProbit, Weibull, and Multistage^*c*^	Exponential 2, Exponential 3, Exponential 4, Exponential 5, Hill, Power, Polynomial^*c*^, and Linear (both constant and modeled variance models for each model above)	Cancer multistage, 1st-order through *n*–1 order, where *n* is the number of dose groups
Distribution assumption	Binomial	Normal	Binomial
^***a***^This control-group SD is the modeled SD. ^***b***^Models selected based on defaults in BMDS and preferences of the U.S. EPA IRIS program (U.S. EPA 2012a). ^***c***^Of order *n*–1, where *n* is the number of dose groups for each data set modeled.			

The BMR levels associated with the batch-calculated BMD/Ls, termed BMD/BMDL_10/1SD_ throughout, were standardized only according to the mathematical representation of the response data (continuous or dichotomous), following the recommendations outlined in BMD Guidance (U.S. EPA 2012a). A 10% BMR was used for dichotomous data, and a “change in the mean equal to one control SD” BMR was used for continuous data. These two BMR levels are the standard reporting levels for each dose–response type and do not necessarily represent equivalent values. However, Crump (1995) found that using a 1 control-group SD change for the continuous end point gives an excess risk of approximately 10% for the proportion of individuals < 2nd percentile or > 98th percentile of controls for normally distributed effects. Tailoring of BMR levels to the specific type or severity of the end point measured may depend on the decision-making context for which the BMD results will be used and was, therefore, beyond the scope of the present study.

The final model and associated BMD and BMDL for each dose–response set was selected according to the following criteria. The Viable model with the lowest Akaike’s information criterion (AIC) was always selected if the BMDLs were “sufficiently close,” that is, there was no more than a 3-fold difference between lowest and highest BMDL for Viable models (Davis et al. 2011). Otherwise, the model with the lowest BMDL was selected. If no models were Viable, the highest dose(s) were removed, and the models were re-run in cases where at least three (including control) doses remained. If two or more models had the same lowest AIC value and the BMD and BMDL values were different, the averages of the BMDs and BMDLs of those models were recorded. This final step is not done automatically by the BMDS Wizard. After completion of a modeling run of a dose–response data set and BMR, we recorded the BMD and BMDL for all successful models as well as any applicable model warnings or notes (based on passing or failing the tests listed in the decision logic reported in Supplemental Material, Table S1). If no model was successful, the dose–response data set was noted as having failed BMD modeling.

*BMD/L selection*. If a chemical had more than one dose–response data set, we selected the BMDs and BMDLs as follows: *a*) the lowest BMD (without warnings, if available) and the BMDL associated with it, and *b*) the lowest BMDL (if different from the previous BMDL). These were selected regardless of end point/effect.

*Data analysis*. We examined the features of the overall resulting data set, including the range and distribution of the batch-processed BMD and BMDL values. BMDs and BMDLs calculated using the method described here were compared to BMDLs and other PODs, particularly NOAELs, for the same chemicals as reported in previous human health assessments, using several linear regression methods to calculate Pearson (*R*^2^ values) and Spearman (ρ values) correlations. Tests for significance were calculated using two-tailed unpaired *t*-tests. The chi-squared test for trend in proportions was used to test for significance in trends. *p*-Values < 0.05 were considered significant. Statistical analysis and graphical outputs were produced by Microsoft Excel (Armonk, NY), R (version 2.15; R Foundation for Statistical Computing, Vienna, Austria), GraphPad Prism (La Jolla, CA) software, and the Health Assessment Workspace Collaborative (Shapiro 2013).

## Results

*Curation of chemicals and data*. We identified 1,260 chemicals with at least one U.S. EPA- or California EPA-derived toxicity value. Mixtures, chemicals missing structural information, and inorganic, organometallic, and duplicate structures were removed during curation (*n* = 374). We collected dose–response data for 352 of the remaining 886 chemicals with toxicity values, yielding 880 dose–response data sets. We prioritized data collection according to public availability of the information (see Supplemental Material, Figure S1).

*BMD modeling*. Of the 880 dose–response data sets available for analysis, we successfully [termed Viable in BMDS Wizard (ICF International)] modeled 603 according to the prespecified statistical and other adequacy criteria given in Supplemental Material, Table S1 without any adjustments. Ninety-nine dose–response data sets contained fewer than three dose groups (including the control) and thus could not be modeled. For 178 dose–response data sets, a first-pass attempt to model with all dose groups failed. When the highest dose group was omitted, we obtained successful models for an additional 66 dose–response data sets while 112 remained unmodelable. In total, 669 dose–response data sets were successfully modeled, whereas 211 dose–response data sets were not (see Supplemental Material, Figure S2). The modeled data sets covered 255 chemicals, whereas dose–response data sets for a remaining 97 chemicals did not pass model fit and completion tests. Overall, the modeling success rates were 86, 91, and 75% for cancer, dichotomous, and continuous data sets, respectively. The most frequently used model was exponential for continuous data sets and log-logistic for dichotomous data sets. See Supplemental Material, Figure S3, for additional information on the models used, including a characterization of the models used by the number of dose groups.

We also evaluated the model-fit warnings associated with successful models (271 of 669, or 40.5%, successful data sets had at least one warning), and we found that the majority (64%) of these concerned extrapolating more than three times below the lowest non-zero dose (median values were 6.4 for BMDL and 5.0 for BMD extrapolations). The next most common (13.2%), but not mutually exclusive, warning was high (> 5) BMD/BMDL ratio (see Supplemental Material, Figure S4).

**Figure 2:**
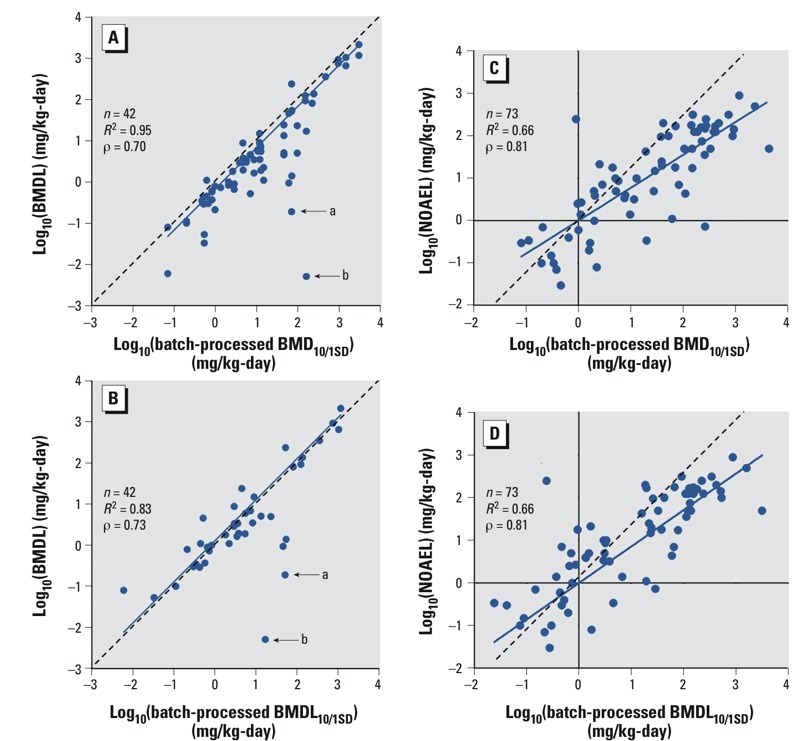
Correlations of batch-calculated BMDs and BMDLs with BMDLs (*A*,*B*) and NOAELs (*C*,*D*) as reported in human health risk assessments. *R*^2^ values represent squared Pearson correlations. ρ Values represent Spearman correlations. Dotted line represents the regression line through the origin. Solid line represents the best-fit line. “a” denotes dichloromethane values; “b” denotes trichloroethylene values.

*Comparison to PODs reported in human health assessments*. We made statistical comparisons among previously reported and batch-calculated PODs for the PODs used as the basis for published RfDs (fewer data were available for comparison of PODs for other toxicity values and analyses were designed to be as consistent as possible). The lowest batch-calculated BMD_10/1SD_ and BMDL_10/1SD_ were compared with BMDLs from the same data set used for PODs in previous human health assessments. We found these untransformed values to be significantly and highly linearly correlated (*R*^2^ of 0.95 and 0.83, respectively, *n* = 42) (Figures 2A,B). More than 88% of values were within one order of magnitude of the BMDLs used in past assessments, and the mean values were not significantly different (see Supplemental Material, Figure S5). We noted two outliers (both were included in the correlation analysis): dichloromethane and trichloroethylene (marked “a” and “b,” respectively, in Figure 2A,B).

These same batch-calculated BMD_10/1SD_ and BMDLs_10/1SD_ were also compared with NOAELs from the same data set used as PODs in previous human health assessments, and, after log-transformation to account for skewness, were found to be significantly linearly correlated (*R*^2^ of 0.66 for both, *n* = 75) (Figure 2C,D; see also Supplemental Material, Table S2, for the list). The comparison was further made with LOAELs used previously as PODs, or all previous PODs aggregated together, with significant linear correlation after log transformation (LOAELs: *R*^2^ of 0.78 and 0.63, respectively, *n* = 20; PODs: *R*^2^ of 0.62 and 0.59, respectively, *n* = 138) (see Supplemental Material, Figure S6).

**Figure 3:**
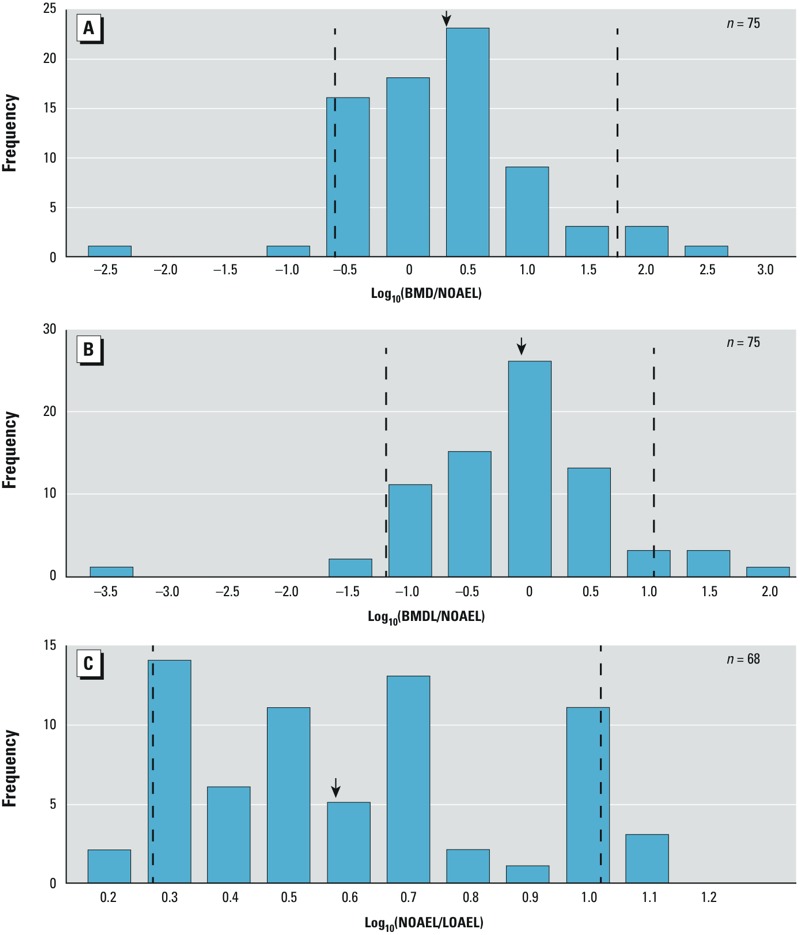
Histograms of log-transformed ratios of batch-calculated BMDs to NOAELs (*A*), BMDLs to NOAELs (*B*), and LOAELs to NOAELs (*C*). The y-axis shows the frequency counts; the x-axis shows the magnitude of the ratio; the dashed lines indicate the 5th and 95th percentiles of the distribution; and the arrows indicate median values.

*Comparison to NOAELs reported in human health assessments*. We calculated the ratios of batch-calculated BMDs_10/1SD_ and BMDLs_10/1SD_ to oral NOAELs reported in previous health assessments (Figure 3A,B; *n* = 75) (there was an insufficient number of inhalation NOAELs for statistical comparison), respectively. The median ratio of BMDs_10/1SD_:NOAEL was 1.96, with a 5th–95th percentile range of 0.24–56.9. The median ratio of BMDLs_10/1SD_:NOAEL was 0.89, with a 5th–95th percentile range of 0.06–23.7. In addition, we compared LOAELs from the studies used to identify the NOAELs used in the previous health assessments when available, and found a median ratio of 3.81 with a 5th–95th percentile range of 1.87–10.7 (Figure 3C, *n* = 68).

**Figure 4:**
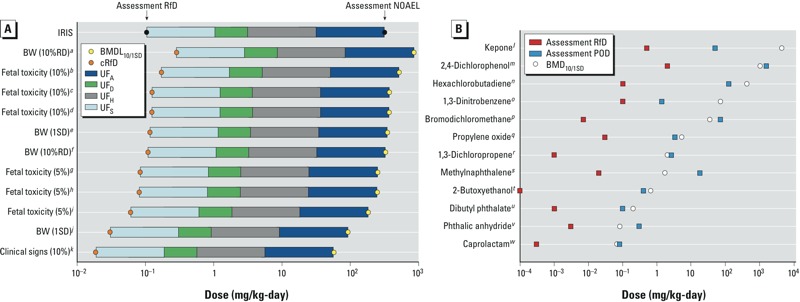
Array of batch-calculated BMDLs for the critical effects observed in studies of nitroguanidine compared with the IRIS NOAEL and RfD (*A*), and array of batch-calculated BMDs for selected chemicals compared with RfDs and PODs reported in human health assessments (*B*). Yellow circles indicate batch-calculated BMDs and BMDLs; orange circles indicate RfDs based on batch-calculated BMDLs. Uncertainty factors: UF_A_, interspecies uncertainty; UF_D_, database incompleteness; UF_H_, intraspecies variability; UF_S_, subchronic to chronic extrapolation. ***^a^***Reduced body weight gain. ***^b^***Retarded ossification of pubis. ***^c^***< 3 sternebrae ossified. ***^d^***< 3 caudal vertebra ossified. ***^e^***Reduced weight gain in female rats. ***^f^***Reduced weight gain in female rats. ***^g^***Retarded ossification of pubis. ***^h^***< 3 caudal vertebra ossified. ***^i^***< 3 sternebrae ossified. ***^j^***Reduced body weight gain. ***^k^***Maternal toxicity. ***^l^***Renal lesions (glomerulosclerosis). ***^m^***Decreased delayed hypersensitivity response. ***^n^***Renal tubule regeneration. ***^o^***Increased splenic weight. ***^p^***Renal cytomegaly. ***^q^***Nest-like infolds of the nasal respiratory epithelium. ***^r^***Chronic irritation. ***^s^***Lung adenoma or carcinoma (combined). ***^t^***Hemosiderin deposition in the liver. ***^u^***Increased mortality. ***^v^***Lung and kidney histopathology. ***^w^***Reduced offspring body weight.

*Batch-calculated BMD/Ls permit comparisons among adverse effects and chemicals*. We selected nitroguanidine (CASRN 556-88-7) as an example chemical to illustrate how the standardized BMD approach can be used to calculate “batch-calculated candidate reference values” among multiple adverse health effects. Several dose–response data sets were available for nitroguanidine, including body weight changes, maternal toxicity, and non-neoplastic histopathological changes. In the original human health assessment, all of these end points were used to select a single NOAEL and derive an RfD. The collection of batch-calculated BMDLs_10/1SD_ was arrayed and compared to the NOAEL (Figure 4A) (U.S. EPA 1993). Uncertainty factors for interspecies uncertainty (UF_A_ = 10), intraspecies variability (UF_H_ = 10), subchronic to chronic extrapolation (UF_S_ = 10), and database incompleteness (UF_D_ = 3) were applied in the original assessment to derive a reference dose of 0.1 mg/kg/day. “Batch-calculated candidate RfDs” based on batch-calculated BMDLs and the same uncertainty factors are presented in Figure 4A. The same type of analysis was conducted for di(2-ethylhexyl)adipate (CASRN 103-23-1) and pentachlorophenol (CASRN 87-86-5) (see Supplemental Material, Figure S7).

We also used BMDs to illustrate comparisons across chemicals because they reflect central estimates of the dose associated with a standardized level of benchmark response based only on the mathematical representation of the response (continuous or dichotomous). We ranked multiple chemicals according to their calculated BMDs_10/1SD_ (i.e., relative potency) in Figure 4B.

*Study design features as a factor in BMD modeling success*. Because about a quarter of the dose–response data sets could not be successfully modeled using the BMD approach (i.e., Unusable or Questionable according to BMDS Wizard), we reviewed study design characteristics that may be associated with success or failure of modeling. Dose–response data sets that were not modeled successfully failed for a variety of reasons, including poorly modeled variance, goodness of fit *p*-test values < 0.05, or a lack of confidence in calculated values, such as by having a BMDL higher than highest dose or a BMD/BMDL ratio > 20 (see Supplemental Material, Figure S8).

**Figure 5:**
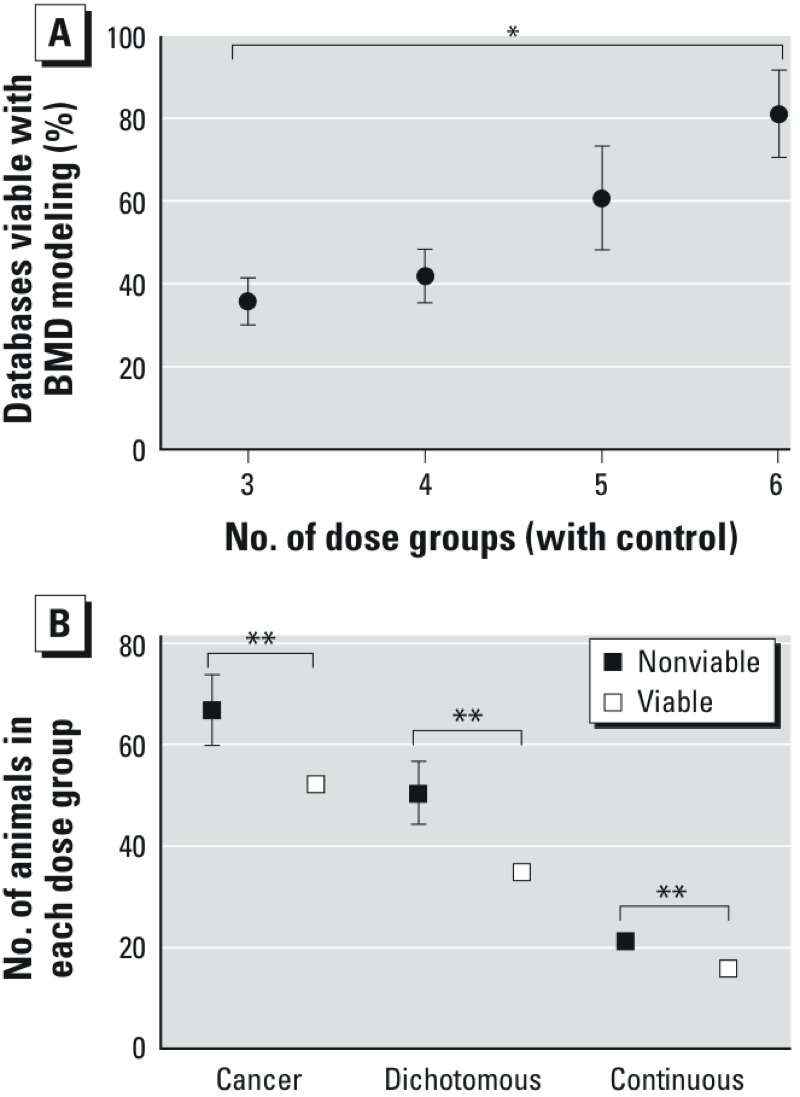
Relationship of Viable BMD models to (*A*) the number of dose groups, (*B*) number of animals in each dose group. Error bars indicate SEs. *p_trend_ < 0.01. **p < 0.001, between group means.

We found a significant (*p* < 0.05) difference in the number of dose groups of successful dose–response data sets versus unsuccessful dose–response data sets (see Supplemental Material, Figure S9). Upon further examination, we observed a significant (*p* < 0.01) trend of increasing viability of models with increasing numbers of dose groups (Figure 5A). We found that the number of animals per dose group is statistically significantly associated with BMD modeling success (*p* < 0.001) (Figure 5B). Successful models had lower numbers of animals per dose group than unsuccessful models, across all dose–response data types (i.e., dichotomous, dichotomous-cancer, continuous). There was no correlation between the number of dose groups and number of animals per dose group (data not shown). The spacing between the dose level of dose group 2 and dose group 3 was not associated with BMD modeling outcome (data not shown).

## Discussion

We evaluated the efficacy and reliability of a standardized BMD approach, compared it to chemical-specific BMD modeling, and identified lessons learned for future application of BMD modeling in human health assessments. Our analysis indicates that a standardized approach can be successfully applied to a large number of chemicals and data sets. We limited our analysis to the dose–response data sets from which PODs were identified in past assessments but which were not necessarily chosen with BMD modeling in mind. It is likely that this approach would be even more successful if applied to data sets specifically chosen for BMD modeling (e.g., those with sufficient dose groups and dose–response trends) (Davis et al. 2011).

We compared batch-calculated BMD/Ls based on a standardized, guidance-driven choice of benchmark responses and models with BMD/Ls based on chemical-specific decisions made by different assessors and at different times. Batch-calculated BMD/Ls were significantly correlated with BMDLs derived one chemical at a time. Approximately 20% of the batch-calculated values used a different BMR from the BMR used in the original assessment (see Supplemental Material, Table S3). Two outliers were dichloromethane and trichloroethylene and the difference was largely due to the use of PBPK model–based dosimetry in the original assessments. The PODs for these two chemicals already reflected a conversion from animal to human equivalent dose and an adjustment for human toxicokinetic variability (U.S. EPA 2011b, 2011c). For trichloroethylene, an additional difference was the use of a 10% extra risk in the batch-calculated modeling as opposed to a 1% extra risk in the assessment (U.S. EPA 2011c).

Because our analysis uniquely included BMD, BMDL, and NOAEL values for 75 chemicals, we evaluated the relationship between batch-calculated BMDs and BMDLs and NOAELs selected during the course of a human health assessment. NOAELs are thought to approximate the dose that represents a 1–5% BMR (Allen et al. 1994a). However, our findings show that BMDs based on a 10% or 1-SD BMR are similar to NOAELs (Figure 3B) (U.S. EPA 2012a). Similarly, Sand et al (2011) found that the median upper bound on extra risk at the NOAEL was approximately 10% using 786 National Toxicology Program cancer data sets.

Our analysis also highlights the utility of BMD modeling and batch-processed candidate reference value calculations in evaluating the entirety of a database on a specific chemical. Although we used only data from the critical study evaluated in the original human health assessment, our findings demonstrate that BMDLs can be calculated in a standardized way to facilitate comparison among multiple health effects and multiple studies at a fixed BMR, consistent with the advice from the National Academies (NRC 2009). This approach also aids identification of outlier evidence or studies if some calculations are orders of magnitude higher or lower than the balance of the data. Thus, this approach can increase objectivity in evaluating multiple studies, enhance transparency, and improve communication with assessors, peer-reviewers, and the general public.

We posit that a comparable approach can be applied in other contexts. For example, high-throughput *in vitro* testing is producing vast amounts of data, consisting of hundreds of dose–response data sets on thousands of chemicals. However, it is unrealistic to expect that individual evaluation of concentration-response relationships in each data set would be commensurate with timely and efficient analyses of these data. Calculation of BMD-like values from *in vitro* data has been suggested (NRC 2009), and our approach can be applied to increase efficiency and transparency in processing such large data sets. Sand et al. (2012) provided a comprehensive review of the considerations for selecting appropriate standardized BMRs when performing concentration-response analysis of *in vitro* data. Consistent selection and application of BMRs and a standardized decision logic yields values that enable comparisons across chemicals (Sirenko et al. 2013) and may inform further testing using a process that is relevant for and familiar to risk managers and decision makers.

In addition, consistently derived BMDs that represent the same biological response can provide valuable quantitative information for other analyses. For example, they can be used to evaluate the potential for quantitative structure-activity relationship modeling. If a chemical structure is found to be predictive of a chemical’s BMD, this would allow decision-makers to evaluate a chemical’s potential hazard to human health even if animal or human data on that chemical are lacking.

Our analysis also informs advancement of a unified dose–response modeling framework that is applied consistently to cancer and noncancer effects proposed by the NRC (2009). The exact nature and implementation of this framework has yet to be determined. For dichotomous end points, current U.S. EPA BMDS guidance specifies a smaller and more constrained set of models for cancer than for noncancer end points (U.S. EPA 2012a). This is a potential area for harmonization as health assessments move towards unifying the cancer and noncancer assessments that could be readily explored by the batch-processing approach explored herein.

Finally, results of our analysis also give insight into study design attributes that increase the potential for BMD modeling success. We observed that successful dose–response data sets tend to have higher numbers of dose groups with fewer animals in each dose group. This result is in accord with Slob et al. (2005) who found, using simulated data, that a higher number of dose groups will help to define the shape of the dose–response relationship and may minimize the risk for unfavorable dose placement. This may be due to several factors. First, as the number of animals in each dose group increases, flexibility in slight deviations between the statistical model’s shape and the true underlying dose–response function decreases. Second, for dichotomous models, there may be sources of variation beyond the binomial statistics assumed by BMDS. In either case, a statistically poor model fit is more likely with more animals per dose group, all other things being equal. This may arise because the test for lack of fit has more power and is more likely to reject the model fit when group sample size is high. Nonetheless, this finding does not imply that fewer animals per dose group is preferable overall. Modeling success needs to be balanced against having enough statistical power to detect a response (Melnick et al. 2008). Because the majority of warnings found in otherwise successful models are due to extrapolation more than three times below the lowest non-zero dose, it is likely that those data sets did not have adequate data to support the BMRs used, and such a warning would not have occurred if a higher BMR has been selected. In addition, the models may not account for nonbiological sources of variation (e.g., group effects) and are dependent on a biological or statistical dose–response trend (Sand et al. 2008). Consideration of these factors together with a more detailed evaluation of the characteristics of dose–response data sets associated with BMD modeling success might illuminate additional useful trends that can inform future study design.

We acknowledge several limitations. Because we did not conduct chemical-by-chemical evaluation, the BMR was not adjusted based on data source or effect severity. A higher or lower BMR may be warranted based on the study type (e.g., epidemiological vs. experimental animal) or severity of the biological response (e.g., developmental malformations vs. organ-specific histopathological changes). However, it is likely that a fixed BMR would be appropriate still (i.e., to enable comparisons among chemicals with the same critical effect and observed severity) in contexts using a standardized BMD process. In addition, BMD models might fit the data mathematically, but may not inform plausibility of the biological response (Davis et al. 2011). Statistical evaluation was limited to model-fit criteria and did not include other considerations such as evaluating the model fit in the low-dose region. Also, cutoffs were fixed in an automated manner according to the decision logic, resulting in less flexibility in assessing model viability than if each cutoff were independently adjusted. These issues can be addressed by a chemical-by-chemical or model-by-model analysis, if necessary.

Furthermore, when using BMD modeling to derive a chemical-specific POD, U.S. EPA guidance recommends an evaluation of the pertinent literature to first identify the most appropriate study(ies) for analysis based on hazard identification, the type of data, and study design (U.S. EPA 2012a). However, our analysis was based on studies that were not necessarily selected for their amenability to BMD modeling. Thus, for a given chemical, it was possible that the dose–response data were unavailable due to inadequate reporting (e.g., original data not provided or only represented graphically in primary literature, group means reported without SDs, no control group reported). This highlights the importance of presenting the raw data used to identify the POD in assessment summaries (such as the online IRIS Summaries).

## Conclusions

Our findings demonstrate that a standardized BMD modeling approach can be used to derive BMD/Ls_10/1SD_ that are significantly and highly correlated with BMDLs derived one chemical at a time. The median ratio of BMDs_10/1SD_ to NOAEL was < 2, whereas BMDLs_10/1SD_ values were generally even lower than NOAELs. Deriving BMD/Ls in a consistent way across chemicals and end points gives values that represent the same response level and which are, therefore, useful in various decision-making contexts, such as identifying a candidate reference value or determining relative potency of chemicals. Such a standardized approach can also be applied to data sets when speed and efficiency are priorities (e.g., *in vitro* assays). Ultimately, our findings show that a standardized approach, which makes BMD modeling transparent and easy to reproduce, is feasible and thus may be considered for wider use in certain decision contexts and types of assessments. In specific cases, expert judgment will still be needed in evaluations of alternative BMRs based on the study type or severity of biological response. Such judgment will assure that the standardized BMD modeling yields an accurate reflection of the underlying biology.

## Supplemental Material

(1.7 MB) PDF
