# Supplementary material for: Standardizing Benchmark Dose Calculations to Improve Science-Based Decisions in Human Health Assessments
Source: Environ Health Perspect. 2014 Feb 25;122(5):499–505. doi: 10.1289/ehp.1307539 (PMC4014768; doi:10.1289/ehp.1307539)
Supplement: (1.7 MB) PDF [file ehp.1307539.s001.pdf]

Environmental Health Perspectives doi:10.1289/ehp.1307539

Supplemental Material

Standardizing Benchmark Dose Calculations to Improve Science-Based Decisions in Human Health Assessments

Jessica A. Wignall, Andrew J. Shapiro, Fred A. Wright, Tracey J. Woodruff, Weihsueh A. Chiu, Kathryn Z. Guyton, and Ivan Rusyn

Table of Contents **Page**

Figure S1. Data and chemical curation process for current study 2

Figure S2. BMD modeling flowchart of the standardized approach as applied in this study 3

Figure S3. Number of models of each type recommended for use in continuous and dichotomous dose-response data sets 4

Figure S4. Types and fraction of all viable model warnings given by BMDS Wizard for viable models 5

Figure S5. Distribution of BMDL values from human health assessments and batch-calculated BMD/Ls 6

Figure S6. Correlations of batch-calculated BMDs and BMDLs with LOAELs and all PODs reported in risk assessments 7

Figure S7. Array of batch-calculated BMDLs compared to IRIS NOAEL and RfD for critical effects of di(2-ethylhexyl)adipate and pentachlorophenol 8

Figure S8. Reasons for model failure across Questionable and Unusable models 9

Figure S9. Number of dose groups for nonviable vs. viable models 10

Table S1. Decision logic for BMDS Wizard 11

Table S2. List of chemicals with batch-calculated BMD/Ls10/1SD and NOAELs 12

Table S3. Comparison between batch processing BMRs and BMRs from original human health assessments 14

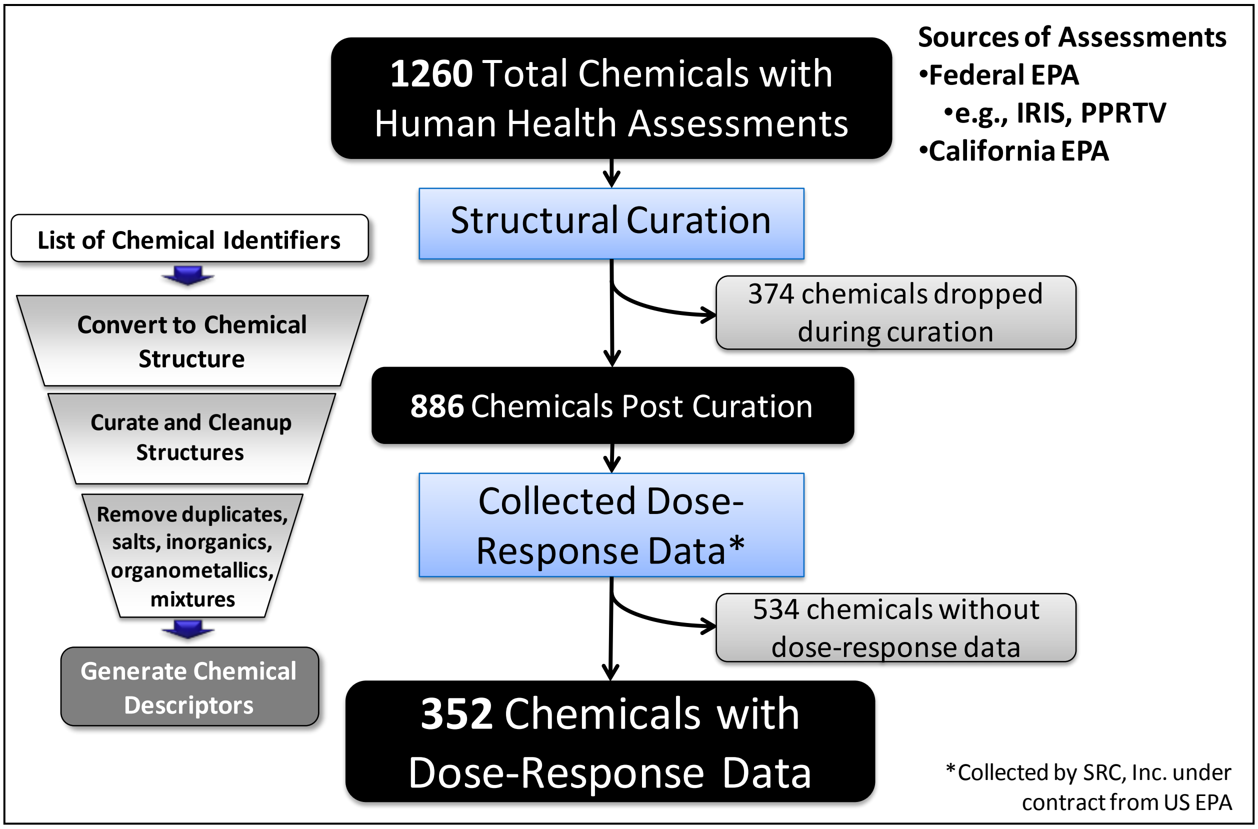

Figure S1. Data and chemical curation process for current study.

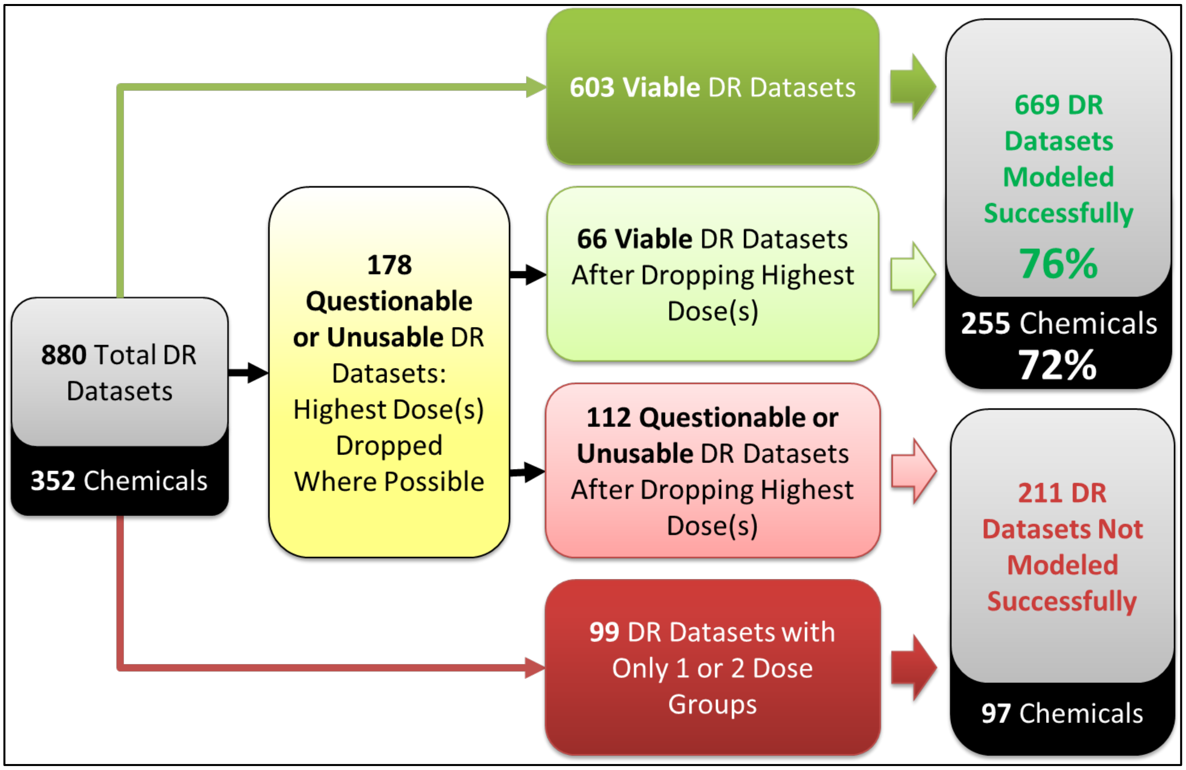

Figure S2. BMD modeling flowchart of the standardized approach as applied in this study.

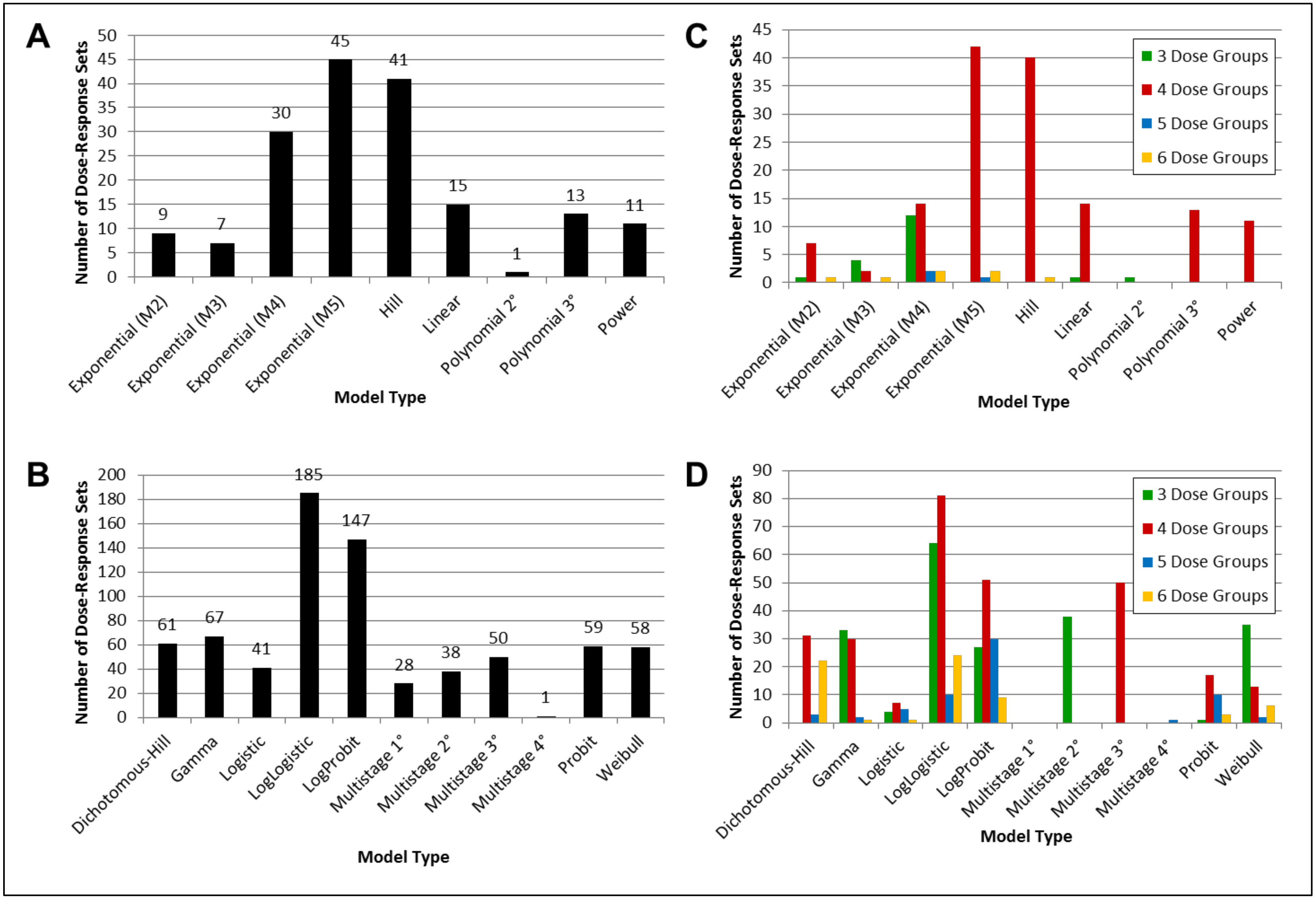

Figure S3. Number of models of each type recommended for use in continuous (A) and dichotomous (B) dose-response data sets; according to number of dose groups for continuous (C) and dichotomous (D) dose-response data sets.

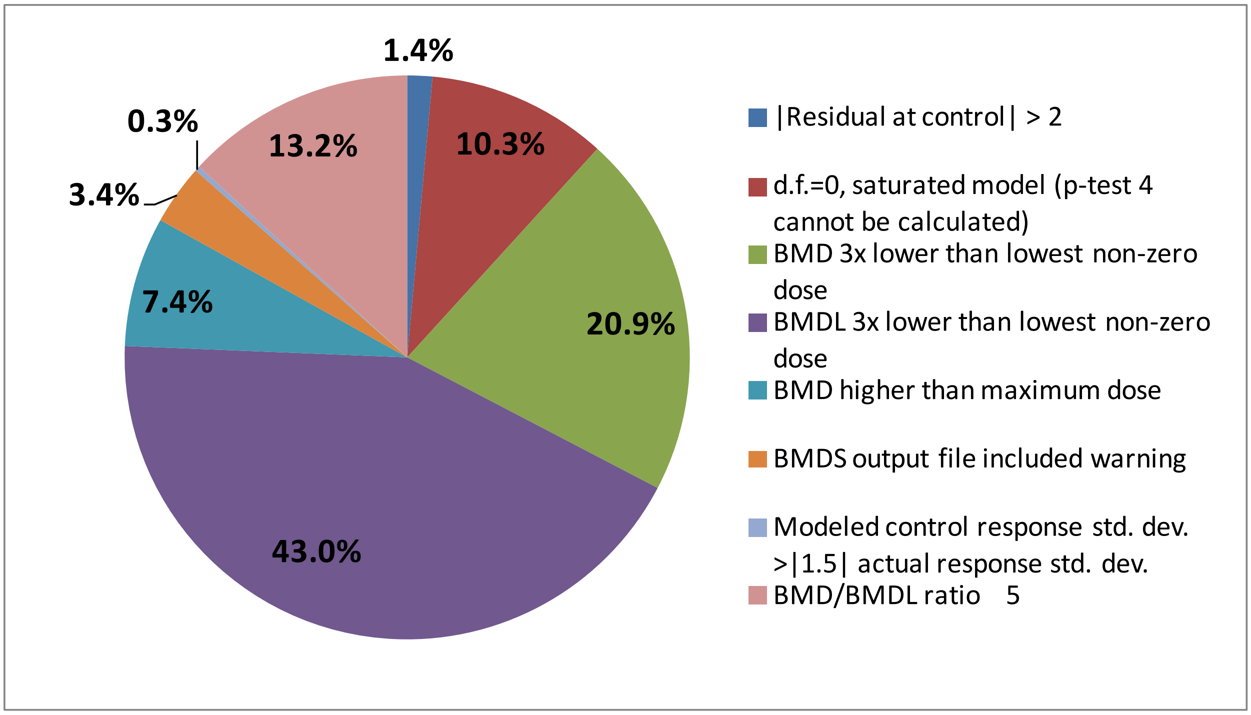

Figure S4. Types and fraction of all viable model warnings given by BMDS Wizard for viable models.

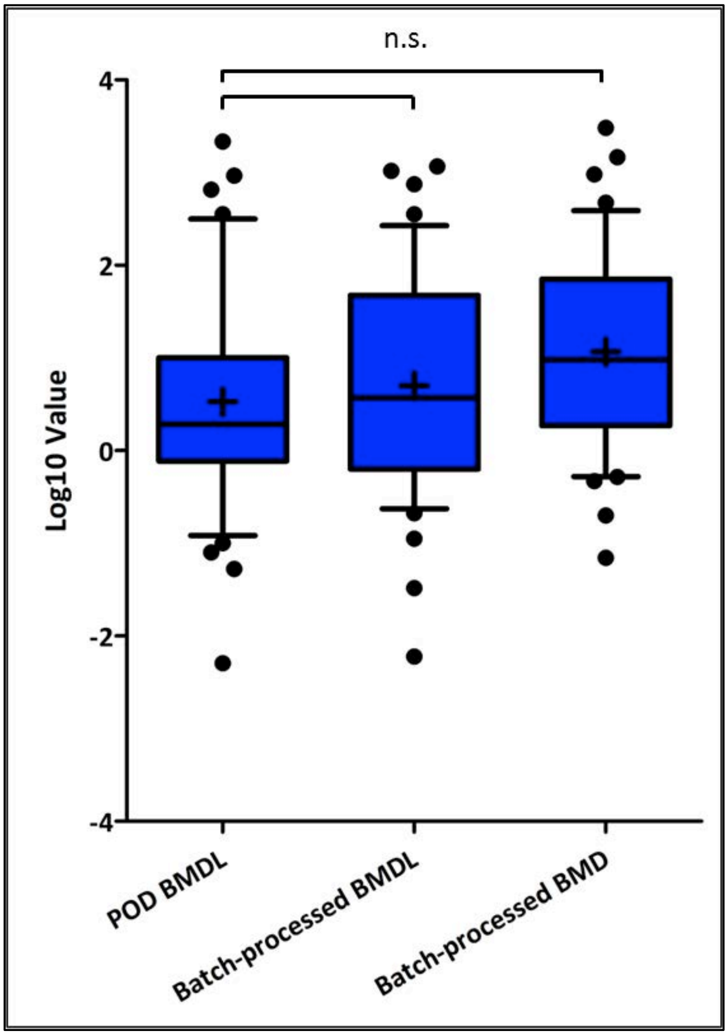

Figure S5. Distribution of BMDL values from human health assessments and batch-calculated BMD/Ls. Whiskers represent 10th to 90th percentile. Plus sign indicates the mean.

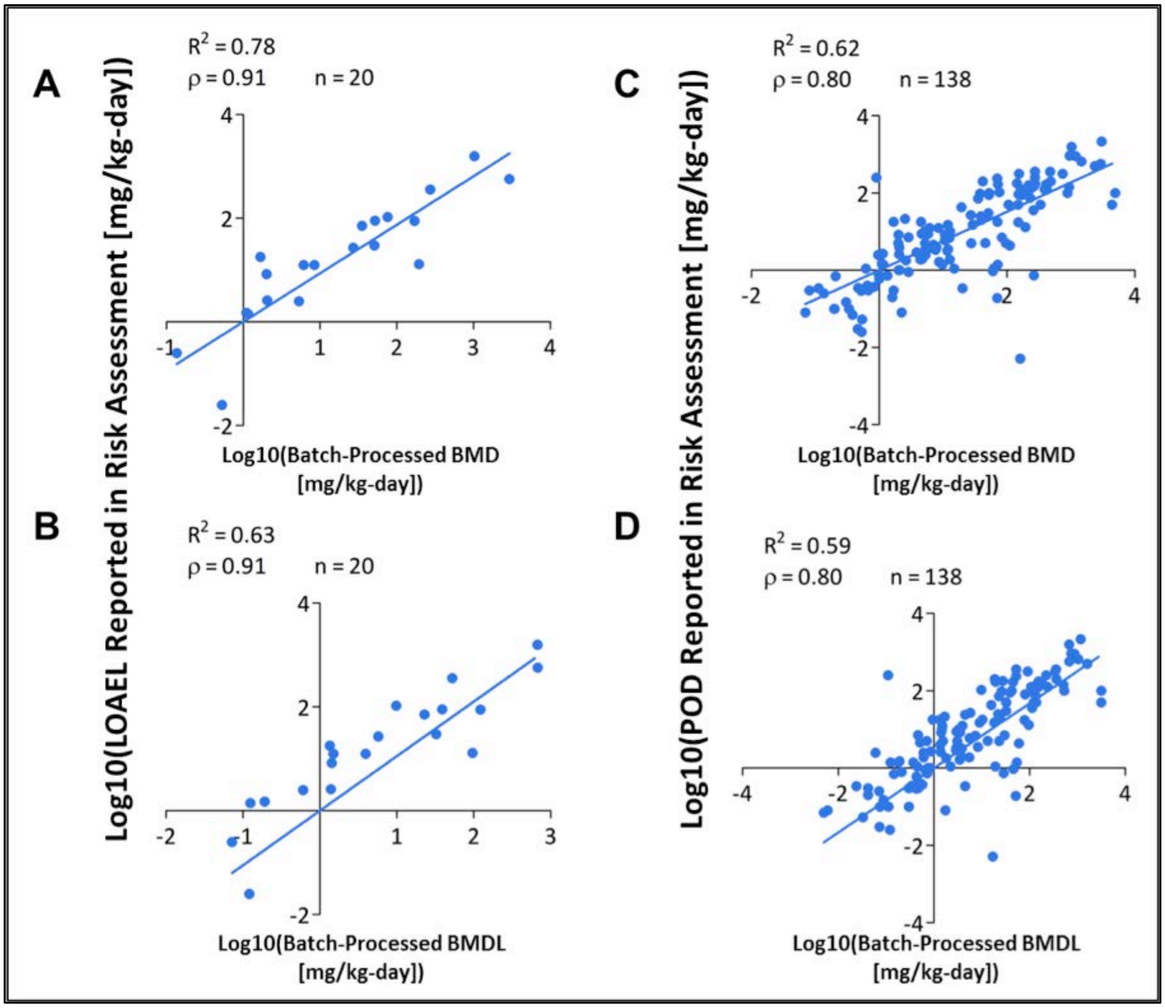

Figure S6. Correlations of batch-calculated BMDs and BMDLs with LOAELs (A,B) and all PODs (C,D) reported in risk assessments. R^2, squared Pearson correlations. ρ, Spearman correlations. Regression line is through the origin.

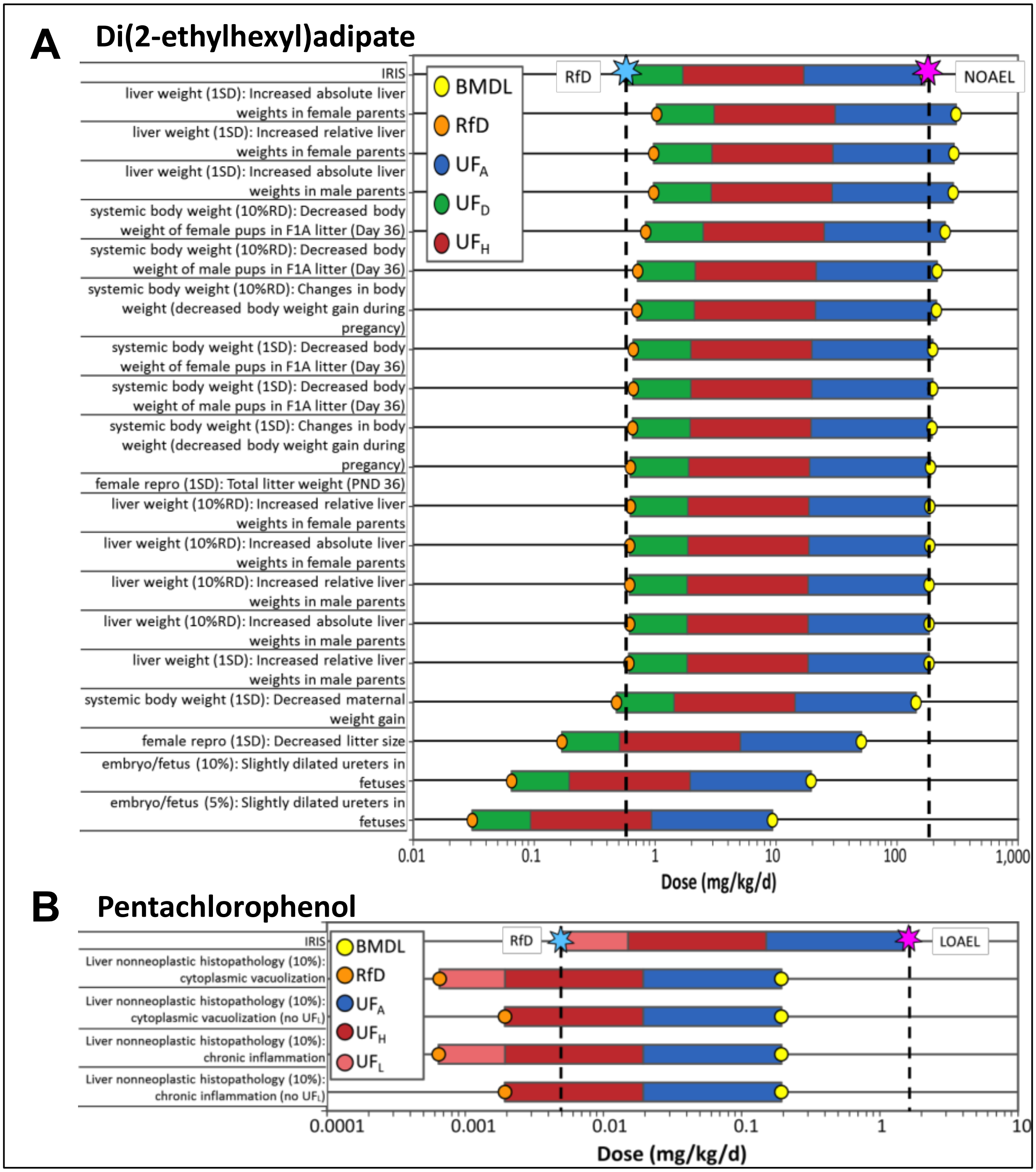

Figure S7. Array of batch-calculated BMDLs compared to IRIS NOAEL and RfD for critical effects of di(2-ethylhexyl)adipate (A) and pentachlorophenol (B). Yellow circles, batch-calculated BMDLs; orange circles, RfDs based on batch-calculated BMDLs; colored bars, uncertainty factors; pink stars, human health assessment PODs; blue stars, human health assessment RfDs.

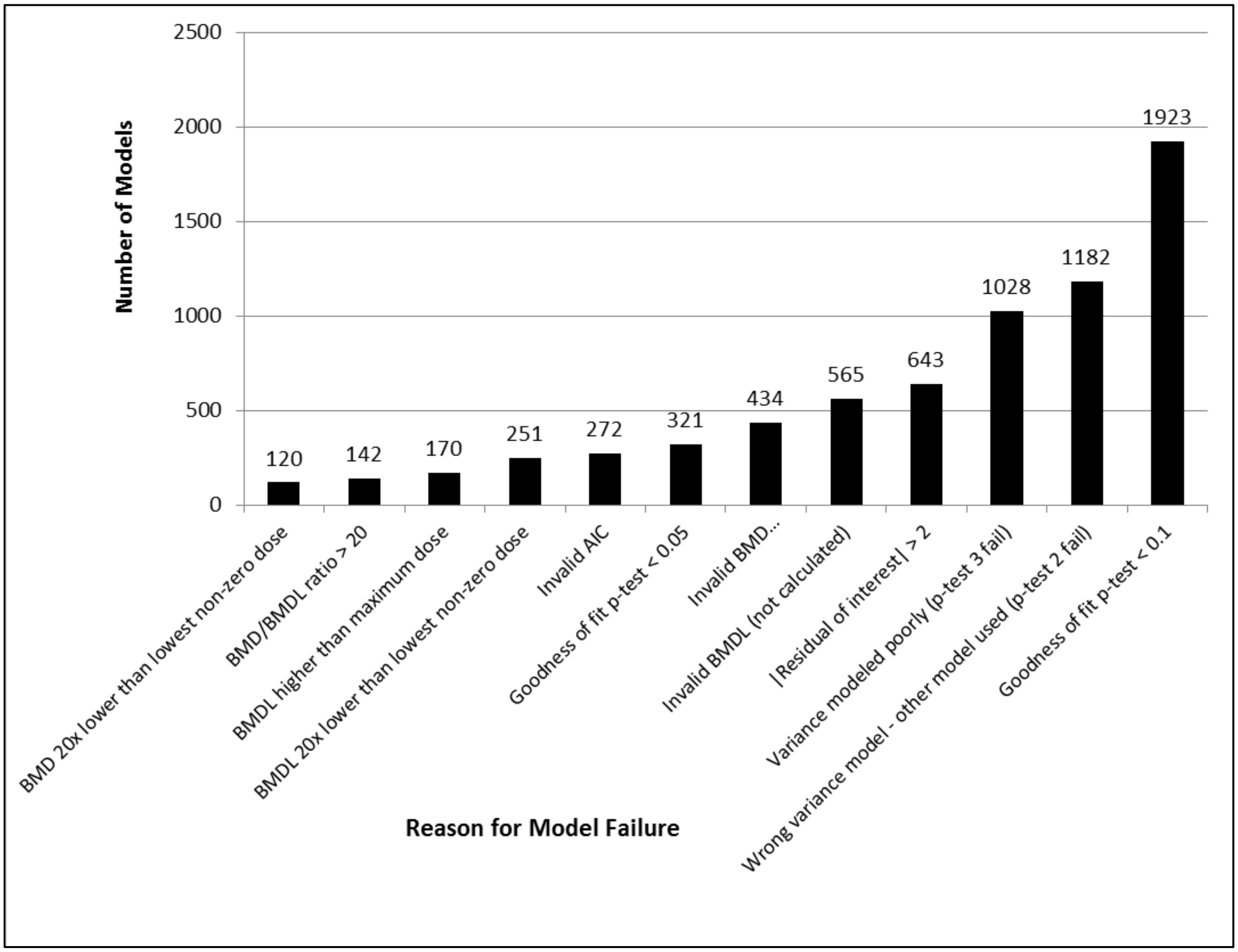

Figure S8. Reasons for model failure across Questionable and Unusable models.

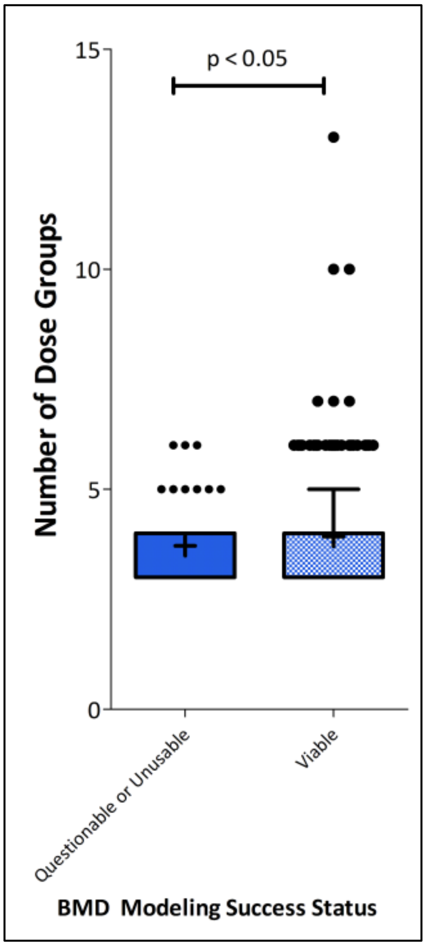

Figure S9. Number of dose groups for nonviable vs. viable models. Whiskers represent 10th to 90th percentile. Plus sign indicates the mean.

Table S1. Decision logic for BMDS Wizard.

Test Description	Test On/Off: Dichotomous	Test On/Off: Dichotomous Cancer	Test On/Off: Continuous	Test Threshold (where appropriate)	Model Category if Test is True	Notes to Show
BMD is not calculated	on	on	on	N/A	Unusable	Invalid BMD
BMDL is not calculated	on	on	on	N/A	Unusable	Invalid BMDL
BMDU is not calculated	off	off	off	N/A	Unusable	Invalid BMDU
AIC is not calculated	on	on	on	N/A	Unusable	Invalid AIC
Wrong variance model	off	off	on	0.1	Unusable	Wrong variance model (p-test 2 fail)
Variance modeled poorly	off	off	on	0.1	Unusable	Variance modeled poorly (p-test 3 fail)
Goodness of fit p-test	on	off	on	0.1	Questionable	Goodness of fit p-test < 0.1
Goodness of fit p-test (cancer)	off	on	off	0.05	Questionable	Goodness of fit p-test < 0.05
Ratio of BMD/BMDL (serious)	on	on	on	20	Questionable	BMD/BMDL ratio > 20
Ratio of BMD/BMDL (caution)	on	on	on	5	Viable (With warning)	BMD/BMDL ratio > 5
Abs(Residual of interest) too large	on	on	on	2	Questionable	|Residual of interest| > 2
BMDS Model Warnings	on	on	on	N/A	Viable (With warning)	BMDS output file included warning
BMD higher than highest dose	on	on	on	1	Viable (With warning)	BMD higher than maximum dose
BMDL higher than highest dose	on	on	on	1	Questionable	BMDL higher than maximum dose
BMD lower than lowest dose (warning)	on	on	on	3	Viable (With warning)	BMD 3x lower than lowest non-zero dose
BMDL lower than lowest dose (warning)	on	on	on	3	Viable (With warning)	BMDL 3x lower than lowest non-zero dose
BMD lower than lowest dose (serious)	on	on	on	20	Questionable	BMD 20x lower than lowest non-zero dose
BMDL lower than lowest dose (serious)	on	on	on	20	Questionable	BMDL 20x lower than lowest non-zero dose
Abs(Residual at control) too large	on	on	on	2	Viable (With warning)	|Residual at control| > 2
Poor control dose std. dev.	off	off	on	1.5	Viable (With warning)	Modeled control response std. dev. >|1.5| actual response std. dev.
d.f. equals 0	on	on	on	NA	Viable (With warning)	d.f.=0, saturated model (p-test 4 cannot be calculated)

Table S2. List of chemicals with batch-calculated BMD/Ls10/1SD and NOAELs.

CAS	Chemical Name
120832	2,4-Dichlorophenol
95943	1,2,4,5-Tetrachlorobenzene
78488	Tribufos
150505	Merphos
96128	1,2-Dibromo-3-Chloropropane
12789036	Chlordane, technical grade
100005	Chloronitrobenzene, p-
2385855	Mirex
105511964	Clodinafop-propargyl
99650	1,3-Dinitrobenzene
100254	Dinitrobenzene, 1,4-
528290	1,2-Dinitrobenzene
84720	Ethyl Carbethoxymethyl Phthalate
32536520	Benzene, 1,1'-oxybis-, octabromo deriv.
115297	Endosulfan
74839	Methyl bromide
99354	1,3,5-Trinitrobenzene
121142	2,4-Dinitrotoluene
121824	RDX
615543	1,2,4-Tribromobenzene
110861	Pyridine
79005	1,1,2-Trichloroethane
118741	Hexachlorobenzene
124481	Dibromochloromethane
135410207	Acetamiprid
75252	Bromoform
106376	1,4-Dibromobenzene
95578	2-Chlorophenol
75865	Acetone Cyanohydrin
175013180	Pyraclostrobin
606202	Dinitrotoluene, 2,6-
110009	Furan
75990	Dalapon
25057890	Bentazone
111911	Bis(2-Chloroethoxy)Methane
109693	1-Chlorobutane
126987	Methacrylonitrile
145701231	Florasulam
50000	Formaldehyde
68122	N,N'-Dimethylformamide

CAS	Chemical Name
58902	2,3,4,6-Tetrachlorophenol
239110157	Fluopicolide
100425	Styrene
140578	Aramite
79622596	Fluazinam
108918	Cyclohexanamine
103231	Di-2-Ethylhexyl Adipate
68157608	Forchlorfenuron
142289	Dichloropropane, 1,3-
8003347	Pyrethrins
1330207	Xylenes (mixed isomers)
2691410	Octogen
131983727	Triticonazole
556887	Nitroguanidine
206440	Fluoranthene
86737	9H-Fluorene
85687	Butyl Benzyl Phthalate
129000	Pyrene
108601	Bis(2-chloro-1-methylethyl) ether
112281773	Tetraconazole
78591	Isophorone
108101	4-methyl-2-Pentanone
83329	Acenaphthene
123308	Aminophenol, p-
111991094	Nicosulfuron
111900	Diethylene Glycol Monoethyl Ether
84742	Dibutyl Phthalate
107211	Ethylene glycol
78831	Isobutyl Alcohol
422556089	Pyroxsulam
100210	Phthalic Acid, p-
67641	Acetone
67561	Methanol
105602	Caprolactam
108054	Vinyl Acetate

Table S3. Comparison between batch processing BMRs and BMRs from original human health assessments.

CAS	Chemical Name	Source	Effect	BMR used in batch processing	Lowest BMD from batch processing	Lowest BMDL from batch processing	Original assessment POD	Effect Level of POD	Original assessment POD BMR	Reason for selection of POD BMR	Assessment POD BMR same as batch processing BMR?
143500	Kepone	IRIS	nonneoplastic histopathology	10%	0.070	0.006	0.08	BMDL10	10%	BMR of a 10% increase in glomerulosclerosis was selected under an assumption that it represents a minimal biologically significant change	same
87683	Hexachlorobutadiene	PPRTV	nonneoplastic histopathology	10%	0.200	0.112	0.1	BMDL	10%	Rationale not provided	same
5436431	2,2',4,4'-Tetrabromodiphenyl ether	IRIS	neurobehavior	1SD	0.470	0.345	0.35	BMDL1SD	1SD	In the case of motor activity, there is no specific change that is generally regarded as indicative of an adverse response. In the absence of some idea of the level of response to consider adverse, the benchmark response (BMR) selected was a change in the mean equal to one SD from the control mean	same
60348609	2,2',4,4',5-Pentabromodiphenyl ether	IRIS	neurobehavior	1SD	0.521	0.425	0.29	BMDL1SD	1SD	A change in the mean equal to 1 SD of the control mean was assumed to represent a minimal biologically significant change	same
79061	Acrylamide	IRIS	nonneoplastic histopathology	10%	0.538	0.033	0.053	BMDL(HED)	5%	A BMR of 5% extra risk was selected for the following reasons: (1) this effect level is considered to be a minimal biologically significant change given the critical effect of degenerative nerve changes; (2) the $BMDL_5$ remained near the range of observation; and (3) the 5% extra risk level is supportable given the relatively large number of animals used in the principal studies	different

CAS	Chemical Name	Source	Effect	BMR used in batch processing	Lowest BMD from batch processing	Lowest BMDL from batch processing	Original assessment POD	Effect Level of POD	Original assessment POD BMR	Reason for selection of POD BMR	Assessment POD BMR same as batch processing BMR?
96184	1,2,3-Trichloropropane	IRIS	organ weight	1SD	0.618	0.448	1.1	BMDL(adj)	10%	BMR of a 10% change in absolute liver weight was selected under an assumption that it represents a minimal biologically significant change	different
88733	Chloronitrobenzene, o-	PPRTV	nonneoplastic histopathology	10%	0.672	0.299	0.3	BMDL	10%	In accordance with the U.S. EPA (2000) BMD methodology, the default benchmark response (BMR) of a 10% increase in extra risk was used as the basis for the BMD (BMD10), with the BMDL10 represented by the 95% lower confidence limit on the BMD10.	same
100016	Nitroaniline, 4-	PPRTV	none	1SD	0.828	0.574	0.37	BMDL	1SD	Although data are available to suggest methemoglobin levels in blood can produce adverse effects in humans, no corresponding data were available for rodents. Therefore, a default benchmark response of 1 SD above the control mean was used to estimate the benchmark dose, as recommended by U.S. EPA (2000).	same
80079	Sulfonylbis(4-chlorobenzene), 1,1'-	PPRTV	nonneoplastic histopathology	10%	0.980	0.212	0.79	BMDL	10%	The default benchmark response of 10% for quantal data	same
67721	Hexachloroethane	IRIS	nonneoplastic histopathology	10%	1.344	0.728	0.728	BMDL10	10%	In this case, a BMR of a 10% increase in the incidence of renal tubule atrophy and degeneration was selected under an assumption that it represents a minimal biologically significant change	same
67663	Chloroform	IRIS	#N/A	10%	2.085	0.832	1	BMDL10	10%	Rationale not provided	same

CAS	Chemical Name	Source	Effect	BMR used in batch processing	Lowest BMD from batch processing	Lowest BMDL from batch processing	Original assessment POD	Effect Level of POD	Original assessment POD BMR	Reason for selection of POD BMR	Assessment POD BMR same as batch processing BMR?
98953	Nitrobenzene	IRIS	hematology	1SD	2.624	1.812	1.8	BMDL1SD	1SD	In this case, a BMR of a 10% change in either of the respiratory effects was selected under an assumption that it represents a minimal biologically significant change.	same
42576023	Bifenox	PPRTV	nonneoplastic histopathology	10%	2.878	0.658	0.9	BMDL	10%	Rationale not provided	same
108985	Benzenethiol	PPRTV	organ weight	1SD	3.853	2.919	2.91	BMDL	1SD	Per EPA policy, in the absence of a biologically relevant benchmark response level (BMR), a default BMR of 1 SD above the control mean is used.	same
98566	Chlorobenzotrifluoride, 4-	PPRTV	clinical chemistry	1SD	4.640	2.963	8.8	BMDL	1SD	Rationale not provided	same
13674878	Tris(1,3-Dichloro-2-Propyl) Phosphate (Tdcp)	ATSDR	nonneoplastic histopathology	10%	4.708	3.813	1.94	BMDL10	10%	In the absence of a clear criteria as to what level of change in kidney weight should be considered adverse, the BMR was defined as a change in mean kidney weight equal to 1 SD from the control mean (EPA 2000)	same
542756	1,3-Dichloropropene, mixed isomer	IRIS	nonneoplastic histopathology	10%	5.241	3.601	3.4	BMDL10	10%	Rationale not provided	same
123739	trans-Crotonaldehyde	PPRTV	nonneoplastic histopathology	10%	5.599	3.001	3.4	BMDL	10%	Rationale not provided	same

CAS	Chemical Name	Source	Effect	BMR used in batch processing	Lowest BMD from batch processing	Lowest BMDL from batch processing	Original assessment POD	Effect Level of POD	Original assessment POD BMR	Reason for selection of POD BMR	Assessment POD BMR same as batch processing BMR?
75354	1,1-Dichloroethylene	IRIS	nonneoplastic histopathology	10%	7.048	0.517	4.6	BMDL10	10%	No exposure-related neoplastic changes occurred at any exposure. No hepatocellular necrosis was evident at any exposure. Based on the minimal nature of the hepatocellular swelling reported by the authors and no change in liver weight, no change in clinical chemistry measurements diagnostic for liver damage, and no other indication of abnormal liver function, the hepatocellular swelling is not considered biologically significant or an adverse effect in this study. The statistically significant hepatocellular midzonal fatty change, however, is considered a minimal adverse effect in this study.	same
109864	2-Methoxyethanol	PPRTV	reproduction	10%	8.660	3.470	1.64	BMDL	10%	In accordance with EPA (2000) guidance, BMDs and BMDLs associated with an extra risk of 10% BMR generally are calculated for all models; a 5% BMR is used for developmental data.	same
110496	Ethylene Glycol Monomethyl Ether acetate	PPRTV	other	10%	8.660	3.470	1.64	BMDL	10%	In accordance with EPA (2000) guidance, BMDs and BMDLs associated with an extra risk of 10% BMR generally are calculated for all models; a 5% BMR is used for developmental data.	same
77474	Hexachlorocyclopentadiene	IRIS	nonneoplastic histopathology	10%	10.665	5.596	6	BMDL10	10%	Rationale not provided	same

CAS	Chemical Name	Source	Effect	BMR used in batch processing	Lowest BMD from batch processing	Lowest BMDL from batch processing	Original assessment POD	Effect Level of POD	Original assessment POD BMR	Reason for selection of POD BMR	Assessment POD BMR same as batch processing BMR?
79345	1,1,2,2-Tetrachloroethane	IRIS	organ weight	1SD	11.850	9.066	15	BMDL1SD	1SD	In this case, a BMR associated with a change of 1 SD from the control mean was selected under an assumption that it represents a minimally biologically significant change	same
91576	2-Methylnaphthalene	IRIS	nonneoplastic histopathology	10%	12.011	8.353	3.5	BMDL5	5%	A benchmark response level of 5% extra risk of the critical effect, pulmonary alveolar proteinosis, was selected for this assessment. This effect is similar to a disorder of unknown etiology that has been identified in humans. If this disorder were to occur in humans following exposure to 2-methylnaphthalene, it is anticipated that children may be more susceptible especially since children affected with the disorder often experience more severe symptoms than adults. Thus, a 5% extra risk of pulmonary alveolar proteinosis was judged to be an appropriate level of extra risk for this critical effect	different
115968	Tris(2-chloroethyl)phosphate	PPRTV	organ weight	1SD	12.542	7.411	6.9	BMDL	1SD	For a dose-dependent increase in relative kidney weight in female rats treated with TCEP for 16 weeks, a default benchmark response (BMR) of 1 standard deviation (SD) from the control mean was used in modeling this endpoint	same
57125	Cyanide ion	IRIS	organ weight	1SD	12.757	5.484	1.9	BMDL1SD	1SD	The BMR for continuous data of 1 SD change in the control mean was selected under the assumption that it represents a minimally biologically significant response level	same

CAS	Chemical Name	Source	Effect	BMR used in batch processing	Lowest BMD from batch processing	Lowest BMDL from batch processing	Original assessment POD	Effect Level of POD	Original assessment POD BMR	Reason for selection of POD BMR	Assessment POD BMR same as batch processing BMR?
99990	Nitrotoluene, p-	PPRTV	nonneoplastic histopathology	10%	14.914	2.239	1.1	BMDL	10%	Rationale not provided	same
156605	trans-1,2-Dichloroethylene	IRIS	other	1SD	45.980	13.322	5.1	BMDL10	10%	In this case, a BMR of 1 standard deviation in spleen cell antibody production was selected under an assumption that it represents a minimal biologically significant change	different
108861	Bromobenzene	IRIS	nonneoplastic histopathology	10%	46.079	4.536	24.1	BMDL10	10%	significant biological response level for the observed spleen effects	same
88722	Nitrotoluene, o-	PPRTV	nonneoplastic histopathology	10%	60.676	46.075	0.94	BMDL	10%	The BMD Lower Confidence Limit for a 10% response (BMDL10) was chosen	same
75092	Dichloromethane	IRIS	nonneoplastic histopathology	10%	70.687	51.424	0.19	BMDL(HED)	1%	based on current BMD guidance (U.S. EPA, 2000).	different
108883	Toluene	IRIS	organ weight	1SD	70.705	52.845	238	BMDL	1SD	Rationale not provided	same
111762	2-Butoxyethanol	IRIS	nonneoplastic histopathology	10%	71.530	54.472	1.4	BMDL(HED)	10%	In this case, EPA concluded a 10% increase in hemosiderin staining, indicating a precursor to an adverse effect, is appropriate for use in deriving the RfD under the assumption that it represents a minimal biologically significant change.	same
591786	2-Hexanone	IRIS	gross pathology	10%	96.894	23.229	5	BMDL10	10%	The POD was defined as the 95% lower confidence limit on the BMD (BMDL) associated with a benchmark response (BMR) of 10% extra risk of axonal swelling. A BMR of 10% is generally used in the absence of information regarding what level of change is considered biologically significant, and also to facilitate a consistent basis of comparison across assessments (U.S. EPA, 2000).	same
108952	Phenol	IRIS	body weight	1SD	152.051	124.591	93	BMDL	1SD	Rationale not provided	same

CAS	Chemical Name	Source	Effect	BMR used in batch processing	Lowest BMD from batch processing	Lowest BMDL from batch processing	Original assessment POD	Effect Level of POD	Original assessment POD BMR	Reason for selection of POD BMR	Assessment POD BMR same as batch processing BMR?
79016	Trichloroethene	IRIS	development	10%	161.322	17.036	0.0051	BMDL01(HED99)	1%	benchmark response (BMR) = 1% extra risk (due to severity of defects, some of which could have been fatal)	different
112345	Diethylene Glycol Monobutyl Ether	PPRTV	hematology	1SD	222.310	81.408	81	BMDL	1SD	In the absence of a biologically relevant response level, the benchmark response (BMR) was chosen to be 1 standard deviation (SD) from the control mean, as recommended by U.S. EPA (2000).	same
104518	Butylbenzene, n-	PPRTV	nonneoplastic histopathology	10%	244.517	136.737	137	BMDL	10%	In accordance with U.S. EPA (2000) guidance, benchmark doses (BMDs) and lower bounds on the BMD (BMDLs) associated with an extra risk of 10% (BMD10 and BMDL10) are calculated for all models	same
111159	Ethylene Glycol Monoethyl Ether Acetate	PPRTV	organ weight	1SD	473.960	356.646	356	BMDL	1SD	The models are run with a BMR of 1 SD from the control mean, as recommended by EPA (2000).	same
109999	Tetrahydrofuran	IRIS	body weight	1SD	955.658	749.906	928	BMDL1SD	1SD	the control mean, as recommended by EPA (2000).	same
78922	Butyl alcohol, sec-	PPRTV	development	1SD	1467.170	1044.000	657	BMD	5%	In the case of pup or fetal body weight, there is no specific decrement that is generally regarded as indicative of an adverse response. Consequently, for each generation, a 5% decrease in the mean pup or fetus body weight per litter (compared with the control mean) was selected as the benchmark response because it was a response rate that fell within the range of experimental dose levels used in the Cox et al. (1975) study.	different

CAS	Chemical Name	Source	Effect	BMR used in batch processing	Lowest BMD from batch processing	Lowest BMDL from batch processing	Original assessment POD	Effect Level of POD	Original assessment POD BMR	Reason for selection of POD BMR	Assessment POD BMR same as batch processing BMR?
71556	1,1,1-Trichloroethane	IRIS	body weight	1SD	3030.650	1166.170	2155	BMDL	10%	A 10% change in mean terminal body weight relative to the control mean was selected as the benchmark response (BMR) level as the minimal level of change generally considered to be biologically significant (U.S. EPA, 2000).	different
